# On protocols and measures for the validation of supervised methods for the inference of biological networks

**DOI:** 10.3389/fgene.2013.00262

**Published:** 2013-12-03

**Authors:** Marie Schrynemackers, Robert Küffner, Pierre Geurts

**Affiliations:** ^1^Systems and Modeling, Department of Electrical Engineering and Computer Science and GIGA-R, University of LiègeLiège, Belgium; ^2^Institute for Practical Informatics and Bioinformatics, Ludwig-Maximilians-UniversityMunich, Germany

**Keywords:** biological network inference, supervised learning, cross-validation, evaluation protocols, ROC curves, precision-recall curves

## Abstract

Networks provide a natural representation of molecular biology knowledge, in particular to model relationships between biological entities such as genes, proteins, drugs, or diseases. Because of the effort, the cost, or the lack of the experiments necessary for the elucidation of these networks, computational approaches for network inference have been frequently investigated in the literature. In this paper, we examine the assessment of supervised network inference. Supervised inference is based on machine learning techniques that infer the network from a training sample of known interacting and possibly non-interacting entities and additional measurement data. While these methods are very effective, their reliable validation *in silico* poses a challenge, since both prediction and validation need to be performed on the basis of the same partially known network. Cross-validation techniques need to be specifically adapted to classification problems on pairs of objects. We perform a critical review and assessment of protocols and measures proposed in the literature and derive specific guidelines how to best exploit and evaluate machine learning techniques for network inference. Through theoretical considerations and *in silico* experiments, we analyze in depth how important factors influence the outcome of performance estimation. These factors include the amount of information available for the interacting entities, the sparsity and topology of biological networks, and the lack of experimentally verified non-interacting pairs.

## 1. Introduction

Networks naturally represent entities such as genes, proteins, drugs or diseases (as nodes) and their mutual relationships (as edges). As immense experimental efforts would be required to comprehensively characterize such networks, computational approaches for network inference have been frequently investigated in the literature. Both unsupervised and supervised approaches have been proposed for network inference. In order to predict interactions, unsupervised inference methods generally derive a score expressing the confidence for a pair of nodes to interact, based on analysis of some experimental data such as gene expression measurements. In contrast to unsupervised methods, supervised approaches additionally require a partial knowledge of the gold standard network. They then exploit some supervised learning algorithm to construct a model that can subsequently be applied to classify the remaining untested pairs. As supervised methods take advantage of known interactions, they can model node specific properties (e.g., in gene regulatory networks, the experimental conditions where a specific regulator becomes active) and thus perform typically much better than unsupervised ones. Supervised learning approaches have been applied to predict several biological networks: protein–protein interaction networks (Yip and Gerstein, 2008; Tastan et al., 2009; Park and Marcotte, 2011), metabolic networks (Yamanishi and Vert, 2005; Bleakley et al., 2007; Geurts et al., 2007), gene regulatory networks (Mordelet and Vert, 2008; Cerulo et al., 2010), epistatic gene networks (Ulitsky et al., 2009; Ryan et al., 2010), or networks of drug-protein interactions (Yamanishi et al., 2008; Bleakley and Yamanishi, 2009; Cheng et al., 2012; Takarabe et al., 2012; Yu et al., 2012).

Performance estimation of both unsupervised and supervised inference methods requires a gold standard of experimentally tested interactions, i.e., pairs of entities labeled as interacting or non-interacting. The validation of supervised methods, however, generally requires special care and the application of cross validation techniques to avoid any sources of bias. Indeed both training and validation need to be performed on the basis of the same partially labeled gold standard. The case of supervised network inference is even more complex as it works on pairs of objects so that the traditional cross validation techniques are not sufficient. In the paper, we propose a critical review of protocols and measures found in the literature for the validation of supervised network inference methods and derive specific guidelines on how to best exploit machine learning techniques for network inference.

The paper is structured as follows. In section 2, we define the problem of supervised network inference and review existing approaches to solve this problem. Section 3 discusses common metrics used to evaluate network predictions (that are common to unsupervised and supervised inference methods). Appropriate ways to perform cross-validation in this context are discussed in section 4. The impact of the lack of negative examples in common biological networks is analyzed in section 5. Finally, section 6 discusses the positive bias on performance induced by the heavy-tailed degree distribution often met in biological networks.

## 2. Supervised network inference

In this section, we first define the problem of supervised network inference more formally and lay out the notations for the rest of the paper. We then briefly review existing approaches to solve this problem.

### 2.1. Problem definition

For the sake of generality, let us assume that we have two finite sets of nodes, *U_r_* = {*n*^1^_*r*_, …, *n^N_U_r__^_r_*} and *U_c_* = {*n*^1^_*c*_, …, *n^N_U_c__^_c_*} of respective sizes *N_U_r__* and *N_U_c__*. A network connecting these two sets of nodes can then be defined by an adjacency matrix *Y* of size *N_U_r__* × *N_U_c__*, such that *y*_*ij*_ = 1 if the nodes *n^i^_r_* and *n^j^_c_* are connected and *y*_*ij*_ = 0 if not. Actually, the subscripts *r* and *c* stand, respectively for *row* and *column*, referring to the rows and columns of the targeted adjacency matrix *Y*. *Y* thus defines a bipartite graph over the two sets *U_r_* and *U_c_*. Standard graphs defined on only one family of nodes, that we call *homogeneous graphs*, can nevertheless be obtained as special cases of this general framework by considering only one set of nodes (i.e., *U* = *U_r_* = *U_c_*). Undirected or directed graphs can then both be represented using a symmetric or an asymmetric adjacency matrix *Y*.

For example, in the case of protein–protein interaction networks, *U_c_* = *U_r_* is the set of all proteins of a given organism and the adjacency matrix is symmetric. A drug-protein interaction network can be modeled as a bipartite graph where *U_r_* and *U_c_* are respectively the sets of proteins and drugs of interest, and element *y*_*ij*_ of *Y* is equal to 1 if protein *n^i^_r_* interacts with drug *n^j^_c_*, 0 otherwise. A regulatory network can be modeled either as a bipartite graph where *U_c_* is the set of all genes of the organism of interest and *U_r_* is the set of all candidate transcription factors (TFs) among them or equivalently by an homogeneous graph and an asymmetric adjacency matrix, where *U_c_* = *U_r_* is the set of all genes and *y*_*ij*_ = 1 if gene *n_i_* regulates gene *n_j_*, 0 otherwise.

In addition, we assume that each node *n* (in both sets) is described by a feature vector, denoted *x*(*n*), typically lying in ℝ^*p*^. For example, features associated to proteins/genes could include their expression in some conditions as measured by microarrays, the presence of motifs in their promotor region, information about their structure, etc. A feature vector *x*(*n_r_*, *n_c_*) can also be associated to each pair of nodes. For example, features directly associated to pairs of proteins could code for the association of the two proteins in another network, their binding in a ChIP-sequencing experiments, etc.

In this context, the problem of supervised network inference can be formulated as follows:

Given a partial knowledge of the adjacency matrix *Y* of the target network in the form of a learning sample of triplets:

$$LS_{p}=\left\{\left(n_{r}^{i_{k}},n_{c}^{j_{k}},y_{i_{k}j_{k}}\right)|k=1,\ldots ,N_{LS}\right\},$$

and given the feature representation of the nodes and/or pairs of nodes, find a function *f* : *U_r_* × *U_c_* → {0, 1} that best approximates the unknown entries of the adjacency matrix from the feature representation (on nodes or on pairs) relative to these unknown entries.

This problem can be cast as a supervised classification problem, with the peculiarity, however, that pairs of nodes, and not single nodes, need to be classified. Next, we discuss existing methods to solve this problem.

### 2.2. Network inference methods

**Figure 1:**
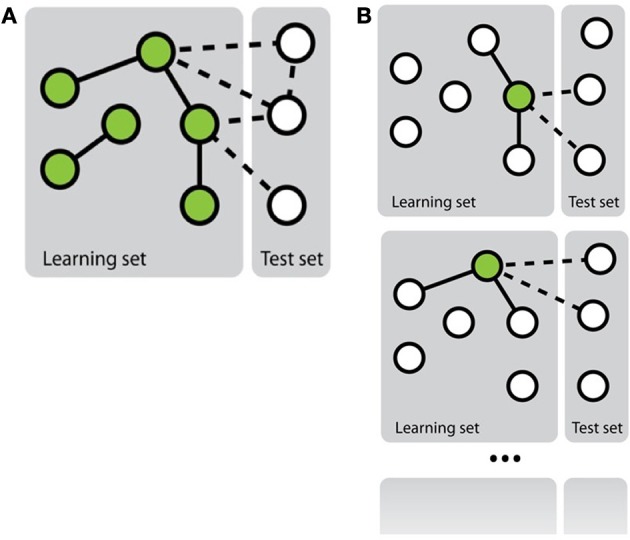
**Schematic representation of the two main approaches to solve the problem of network inference. (A)** The global approach that solves a single supervised learning problem by considering each pair as an object for the learning. **(B)** The local approach that solves several supervised learning problems, each defined by a different node.

Mainly two approaches have been investigated in the literature to transform the network inference problem into standard classification problem (Vert, 2010) (see Figure 1). The first, more straightforward, approach, called *pairwise* or *global*, considers each pair as a single object and then apply any existing classification method on these objects (e.g., Takarabe et al., 2012). This approach requires a feature vector defined on pairs. When features on individual nodes are provided, they thus need to be transformed into features on pairs (Tastan et al., 2009). Several approaches have been proposed in the literature to achieve this, ranging from a simple concatenation or addition of the feature vectors of the nodes in the pair (Chen and Liu, 2005; Yu et al., 2012) to more complex combination schemes (Yamanishi et al., 2008; Maetschke et al., 2013). Different classification methods have been exploited in the literature: nearest neighbor algorithm (He et al., 2010), support vector machines (Paladugu et al., 2008), logistic regression (Ulitsky et al., 2009), tree-based methods (Wong et al., 2004; Yu et al., 2012), etc. In particular, in the context of support vector machines, several kernels have been proposed to compare pairs of objects on the basis of individual features defined on these objects that have been applied for supervised network inference (Vert et al., 2007; Hue and Vert, 2010; Brunner et al., 2012).

In the second approach, called *local* (Mordelet and Vert, 2008; Bleakley and Yamanishi, 2009; Vert, 2010; van Laarhoven et al., 2011; Mei et al., 2013), the network inference problem is divided into several smaller classification problems corresponding each to a node of interest and aiming at predicting, from the features, the nodes that are connected to this node in the network. More precisely, each of these classification problems is defined by a learning sample containing all nodes that are involved in a pair with the corresponding node of interest in *LS*_*p*_. Interestingly, when trying to make a prediction for a given pair (*n^i^_r_*, *n^j^_c_*), one can aggregate the predictions of two classifiers: the one trained for *n^i^_r_* and the one trained for *n^j^_c_*. Note that it is only possible to train a classifier for a node that is involved in at least one positive and one negative interaction in *LS*_*p*_. This prevents the use of the local approach to predict interactions for pairs where both nodes do not satisfy this property. Like for the global approach, in principle, any classification method can be used to train each of the classification models, but mainly support vector machines have been investigated in this context (Mordelet and Vert, 2008; Bleakley and Yamanishi, 2009).

From experiments in the literature, there does not seem to be a clear winner between the local and the global approach in terms of predictive accuracy. The global approach is typically more flexible as it can handle any kinds of features and can make prediction for pairs of unseen nodes, but it requires more computing times and resources, given that it aims to infer a network in one step.

Besides the global and local approaches that make use of existing classification methods, other more specific approaches have also been proposed for supervised network inference. For example, Kato et al. (2005) formulate the problem as a matrix completion problem (with input features) and solve it using an expectation-maximization-based approach. The problem has also been formulated as a distance metric learning problem (Vert and Yamanishi, 2005; Yamanishi, 2009): nodes of the graph are embedded into some euclidean space where they are close as soon as they are connected in the training graph and a mapping is then learned from the node feature space to this euclidean space. A related approach consists in defining a kernel between the nodes in the network that similarly encodes the connections between the nodes in the training graph and then exploit the kernel trick at the output of a regression method to learn an approximation of this kernel from the node features. This framework has been implemented using tree-based ensemble methods (Geurts et al., 2007) and ridge regression (Brouard et al., 2011) for example.

While our brief review focused on the inference of the network from node features, it is also possible to solve this problem by exploiting only the network itself. For example, Cheng et al. (2012) derive a similarly measure between nodes from the network topology and then use this similarity to infer new interactions. In a hybrid approach, some authors have also included features derived from the (training) network topology in the global approach to improve network inference (Ulitsky et al., 2009).

## 3. Evaluation measures

In this section, we review and discuss evaluation measures that have been used to quantify the quality of the predictions given by network inference methods. We focus here on statistical measures that compare a predicted network (or subnetwork) with the true one, as in the case of supervised network inference, some part of the true network is supposed to be available for training. In the general context of network inference, other performance measures have been proposed based either on functional annotations shared by genes/proteins or on topological properties of the inferred networks (see Emmert-Streib et al., 2012, for a survey).

The prediction given by a network inference method for a given pair of nodes can typically be of two kinds: a binary (0–1) value, coding for the presence or the absence of an interaction between the two nodes in the predicted network, or a real value, representing some confidence score associated to the pair: the higher the score, the higher the confidence or certainty of the model that there is an interaction between the nodes in the pair. Depending on the supervised network inference method used, this confidence score can have a probabilistic interpretation or not, but we will not assume it is the case. Of course, one can always transform a confidence score into a binary prediction using a decision threshold. The choice of an appropriate threshold is, however, not an easy problem in practice.

In this section, we assume that we have an adjacency matrix (of a complete or a partial graph) and an equivalent matrix of the binary or real scores predicted by a network inference method. In both cases, our goal is to quantify the quality of the predictions with respect to the true network represented by the adjacency matrix. Protocols to obtain these matrices will be discussed in section 4. We first discuss the case of binary predictions and then compare the receiver operating characteristic (ROC) curves and precision-recall (PR) curves that have been predominantly used to evaluate network inference methods that provide confidence scores. We end the section with a brief survey of other measures and a general discussion.

### 3.1. Binary predictions

Common criteria to evaluate binary predictions are the accuracy (the number of correctly predicted pairs divided by the total number of pairs) or equivalently the error rate (one minus the accuracy). However, network inference problems typically correspond to highly imbalanced classification problems as non-interacting pairs often far outnumber interacting ones. Accuracy is not appropriate in such situations because it greatly favors the majority class (high accuracy is given to a model predicting all pairs as non-interacting pairs). Alternative measures requires to differentiate between the possible types of errors, that are usually counted and compiled in a confusion matrix. In the case of binary classification, this matrix is a 2 × 2 matrix where the columns and rows represent, respectively the actual and the predicted classes and each cell contains the number of pairs corresponding to these classes. Denoting by positive an interaction and by negative a non-interaction, the confusion matrix is as follows:

**Table:** 

	actual positive (*P*)	actual negative (*N*)
predicted positive (pred*P*)	true positive (*TP*)	false positive (*FP*)
predicted negative (pred*N*)	false negative (*FN*)	true negative (*TN*)

Several metrics can be then derived from this matrix to evaluate the performance of a model, among which:

- the *true positive rate* (TPR), also called the *sensitivity* or the *recall*, is equal to the number of true positives divided by the number of actual positives: $\frac{TP}{TP+FN}$ or $\frac{TP}{P}$,
- the *true negative rate* (TNR), also called the *specificity*, is equal to the number of true negatives divided by the number of actual negatives: $\frac{TN}{FP+TN}$ or $\frac{TN}{N}$,
- the *false positive rate* (FPR), corresponding to 1-*specificity*, is equal to the number of false positives divided by the number of actual negatives: $\frac{FP}{FP+TN}$ or $\frac{FP}{N}$,
- the *false negative rate* (FNR), also called the *miss*, is equal to the number of false negative divided by the number of actual negatives: $\frac{FN}{TP+FN}$ or $\frac{FN}{P}$,
- the *precision* is equal to the number of true positives divided by the number of predicted positives: $\frac{TP}{TP+FP}$.
- the *rate of positive predictions* (RPP) is equal to the number of predicted positive divided by the total number of examples: $\frac{TP+FP}{P+N}$ or $\frac{\text{pred}P}{P+N}$
- the *F-score* is equal to the harmonic mean of precision and recall:
  $$F=2\cdot \frac{\text{precision}\cdot \text{recall}}{\text{precision}+\text{recall}}$$

Except for the *F-score*, these measures should be combined to give a global picture of the performance of a method, e.g., sensitivity and specificity or precision and recall. In the case of confidence scores, all these performance measures can be computed for a given threshold on the confidence scores. Nevertheless, often, one would like to measure the performance of a method independently of the choice of a specific threshold. Several curves are used for that purpose that are exposed below.

### 3.2. ROC curves

**Figure 2:**
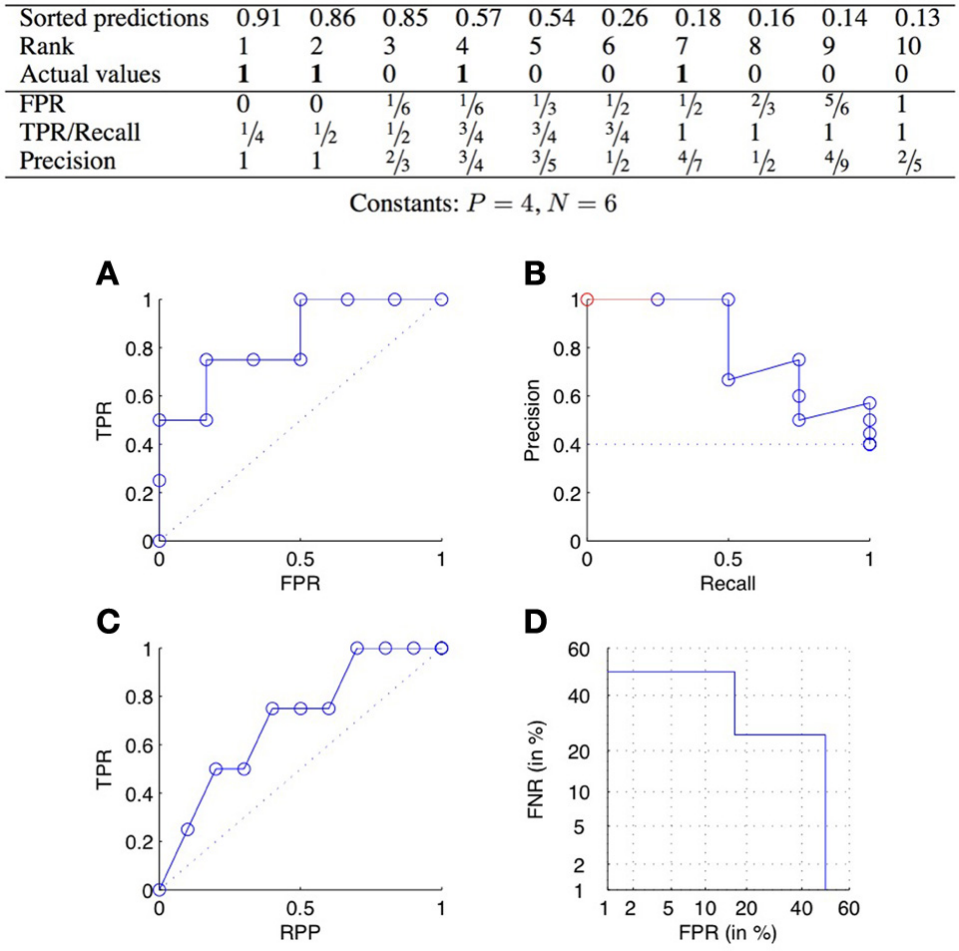
**ROC curve **(A)**, precision-recall curve **(B)**, lift chart **(C)**, and DET curve **(D)** for the scores of the table above**.

ROC curves plot the TPR as a function of the FPR, when varying the confidence threshold (Fawcett, 2006). In concrete terms, the predictions are sorted from the most confident to the least confident, and the threshold is varied from the maximum to the minimum confidence score. Each value of the threshold corresponds to a different confusion matrix, and thus a different pair of values of the TPR and FPR, and corresponds to a point of the ROC curve. See Figure 2A for an example.

The two ends of the curve are always the two points (0, 0) and (1, 1), corresponding, respectively to pred*P* = 0 and pred*P* = *P* + *N*. A perfect classifier would give the highest values of prediction to the pairs that truly interact, and then would have a corresponding ROC curve passing through the point (0, 1). The curve relative to a random classifier corresponds to the diagonal connecting the two points (0, 0) and (1, 1) (the dotted line in Figure 2A).

For comparison purposes, it is often convenient to summarize a ROC curve with a single real number. The most common such measure is the area under the ROC curve (AUROC), which is equal to 1 for a perfect classifier and 0.5 for a random one. On the face of it, one typically assumes that the higher the AUROC, the better the predictions.

In many network prediction tasks, however, the number of interactions is much lower than the number of non-interactions. It is therefore important to achieve a low *FPR* as even moderate *FPR* can easily lead to much more *FP* predictions than *TP* predictions, and hence a very low precision. To better highlight the importance of small *FPR*, partial AUROC values are sometimes used instead of the full AUROC. For example, Tastan et al. (2009) propose statistics like *R*50, *R*100, *R*200, and *R*300 that measure the area under the ROC curve until reaching a *FP* equal to 50, 100, 200, and 300, respectively.

Another summary statistic of a ROC curve is the Youden index (Fluss et al., 2005), which is defined as the maximal value of TPR − FPR over all possible confidence thresholds. It corresponds to the maximal vertical distance between the ROC curve and the diagonal. The Youden index ranges between 0 (corresponding to a random classifier) and 1 (corresponding to a perfect classifier). This statistic was used for example in Hempel et al. (2011) to assess gene regulatory network inference methods.

### 3.3. Precision-recall curves

PR curves plot the precision as a function of the recall (equal to the TPR), when varying the confidence threshold. See Figure 2B for an example. A perfect classifier would give a PR curve passing through the point (1, 1), while a random classifier would have an average precision equal to $\frac{P}{P+N}$ (dotted line in Figure 2B). All PR curves end at the point (1, $\frac{P}{P+N}$) corresponding to predicting all pairs as positive. When all pairs are predicted as negative, recall is 0 but the precision is actually undefined. The coordinates of the first point of the PR curve will therefore be ($\frac{1}{P}$, 1) if the most likely prediction is actually positive, and (0, 0) otherwise. To make all PR curve defined on the full [0, 1] interval, one sometimes adds a pseudo point to the curve at (0, 1) (Figure 2B).

The PR curve is also often summarized by the area under the curve (AUPR). The AUPR is sometimes called MAP, for Mean Average Precision (Manning et al., 2009; Tastan et al., 2009). Like for the AUROC, one typically assumes that the higher the AUPR, the better is the classifier, with the AUPR of a perfect classifier equal to 1 and the AUPR of a random classifier close to $\frac{P}{P+N}$. In practice, the AUPR can be computed from the curve completed with the additional pseudo-point or not. In the second case, one can rescale the area by dividing it by 1 − $\frac{1}{P}$ so that its values is equal to 1 for a perfect classifier. Note that it is important to report exactly on which approach was used to compute the AUPR as it can make a significant difference when the number of positives is very small. For example, the AUPR of the PR curve of Figure 2B is equal to 0.81, 0.75, and 0.56, respectively with the pseudo-point, without the pseudo-point but with rescaling, and without the pseudo-point and without rescaling.

### 3.4. Comparison of ROC and PR curves

**Figure 3:**
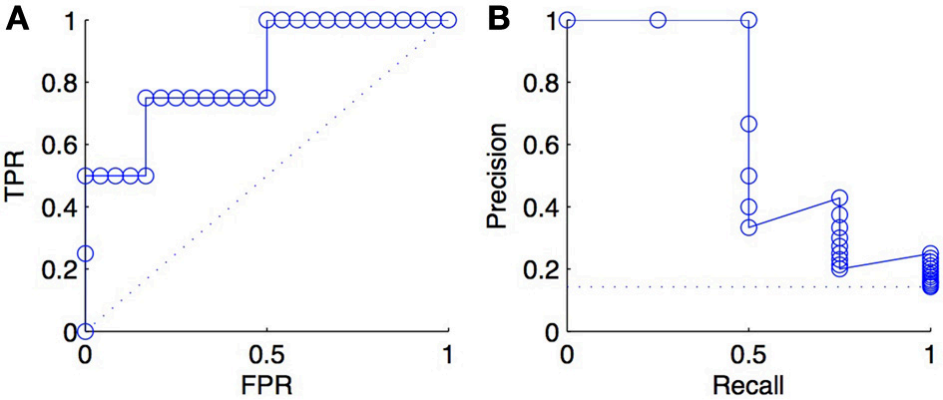
**ROC curve (A) and PR curve (B) for a list of scores where negative examples were tripled with respect to scores of Figure 2**. The comparison with the curves in Figure 2 shows that the ROC curve is unchanged and that the PR curve degrades, as a consequence of tripling the negatives.

An important difference between ROC and PR curves is their different sensitivities to the ratio between positives and negatives (class imbalance) among the tested pairs: a ROC curve is independent of the precise value of this ratio, while a PR curve is not. To illustrate this fact, we triplicated every negative examples in the ranked list of predictions of Figure 2 and plotted the new ROC and PR curves in Figure 3. As expected, we obtained exactly the same ROC curves, while the PR curves are different. This happens because, at fixed recall, a large change in *FP* will lead to no change in the *FPR* used in ROC curves (because to total number *N* of negatives will increase in the same proportion), but to a large change in the precision used in PR curves (Davis and Goadrich, 2006).

This independence with respect to the particular content of the test sample in terms of positives and negatives is actually the main advantage of the ROC curve over the PR curve when it comes to compare different classification methods (Fawcett, 2006). ROC curves allow to compare classification methods whatever will be the ratio between positives and negatives expected when practically applying the model. Because of this independence, however, ROC curves do not really emphasize a particular intervals of values of this ratio and therefore favor methods that are good for a large range of such values. If one knows for example that the ratio between positives and negatives will be very low when applying the classification model, then one is typically only interested in the bottom-left part of the ROC curve. PR curves, on the other hand, provide a better picture of the performance of a method when the ratio between positives and negatives in the test data is close to the ratio one expects when practically applying the model.

The dependence of the PR curve on the ratio between positives and negatives can also be seen as a drawback. First, it means that PR curves (and their associated AUPR) obtained from different datasets can not really be compared when the ratio $\frac{P}{N}$ is very different. This is a limitation if one wants to compare the performance of a method across several networks for example. Second, because of this dependence, it is important that the ratio of positive and negative interactions in the subset of pairs used to validate the method is representative of the final application of the method. Otherwise, the PR curve will not provide a realistic evaluation of the method. Note, however, that it is possible to adapt a given PR curve to a ratio between positives and negatives different than the one adopted to generate it (Hue et al., 2010). Mathematical details are given in the supplementary information.

Another drawback of the PR curve is the potential unstability of the precision for small recall values. Indeed, for small values of predP, the vertical changes of the curve from one confidence threshold to the next can be very huge, independently of the size of the dataset. This is more noticeable when the value of *P* is small because the horizontal changes are then also relatively large. This unstability makes the estimation of the true PR curve highly imprecise (Brodersen et al., 2010). It is, however, actually a direct consequence of the stronger focus put by the PR curve on the top of the ranking with respect to the ROC curve.

Despite these differences, it is interesting to note that a deep connection exists between the ROC and the PR spaces, in that a model dominates another model in the ROC space if and only if it dominates the same model in the PR space (Davis and Goadrich, 2006). In practice, however, it is often the case that a model does not dominate another model over the whole ROC and PR spaces and it might thus happen that a method's AUROC is greater than another method's AUROC, while the opposite is true concerning the AUPR.

### 3.5. Other measures and curves

ROC curves and PR curves are the most popular ways to estimate the performance of biological network inference methods, but some other measures and curves can also be found in the literature.

*Lift charts* (or cumulative lift charts), often used in marketing (Witten and Frank, 2005), plot the TPR, or recall, as a function of the RPP (rate of positive predictions), when varying the confidence threshold. See Figure 2C for an example. A perfect classifier would give a curve going through the points (0, 0), ($\frac{p}{p+n}$, 1) and (1, 1), while a random classifier would be equal to the diagonal connecting the two points (0, 0) and (1, 1).

For example, Geurts (2011) used a lift chart to evaluate the performance of supervised methods for the prediction of regulatory networks, and Yabuuchi et al. (2011) for the prediction of compound-protein interactions. Lift charts explicitly show the number of positive predictions (expressed as a percentage of all possible interactions) that one needs to accept to retrieve a given percentage of all truly positive interactions (recall). This is an important information when one is looking at the experimental validation of the predictions: a method that dominates another in terms of lift chart would require to experimentally test less interactions to achieve a given recall.

Note that when the number of positive examples is much smaller than the number of negative ones, as it often happens in biological networks, there is not much difference between the ROC curve and the lift chart.

*Detection error tradeoff (DET)* curves plot the two types of errors versus each other, i.e., FNR as a function of FPR (Martin et al., 1997). In addition, the two axes are log scaled. An example of DET curve is given in Figure 2D. Without the axis rescaling, a DET curve would be equivalent to a ROC curve (because FNR = 1 − TPR). The interest of the log scale is to expand the lower left part of the curve (which corresponds to the upper left part of the corresponding ROC curve), which as argued in Martin et al. (1997) makes the comparison between different methods easier. DET curves were used in Brunner et al. (2012) to evaluate classification methods working on pairs of objects.

Several authors (Li et al., 2009; Junaid et al., 2010; Lapins and Wikberg, 2010; Niijima et al., 2011) use a *correlation coefficient* for the evaluation of the performance of network inference methods. In this context, the latter is defined as

$$Q^{2}=1-\frac{\sum_{i=1}^{n}{\left(y_{i}-{\hat{y}}_{i}\right)}^{2}}{\sum_{i=1}^{n}{\left(y_{i}-\bar{y}\right)}^{2}}$$

where the sum runs over all tested pairs, *y_i_* and *ŷ_i_* are the true and predicted value corresponding to the *i*th pair and ȳ is the average value of *y_i_*. *Q*^2^ values vary between 0 and 1, with *Q*^2^ = 1 for a perfect classifier.

The *average normalized rank* is another way to compare the performance of different classifiers (Karni et al., 2009; Geurts, 2011). It computes the average rank of all actual positives in the ranking of all pairs according to their confidence score, and then divide it by the total number of pairs. Obviously smaller is the average rank and better is the model.

### 3.6. Discussion

Biological network inference problems, as binary classification problems, are usually substantially imbalanced in favor of the negative class, as the proportion of interacting pairs among all possible pairs is very small. Given the discussion in section 3.4, this speaks in favor of the PR curve over the ROC curve. Let us nevertheless consider three typical scenarios related to the use of supervised network inference techniques and discuss the most appropriate use of these measures in each of these scenarios:

- *Development of new supervised network inference methods:* when trying to design a new supervised network inference method, one needs to assess its performance against existing methods, either on a specific target biological network if the method is specialized or on several networks if the method is generic. In this scenario, one has typically no specific application of the method in mind and the combination of both ROC and PR curves can be a good idea. While AUROC and AUPR summary values can be used for comparison purpose, it is always useful to actually report full ROC and PR curves to better characterize the areas of the ROC and PR where the new method dominates competitors.
- *Prioritizing interactions for experimental validation:* From a ranking of all the pairs from the most likely to interact to the less likely to interact, a biologist may want to validate experimentally the top-ranked pairs, i.e., the potentially new interacting pairs. More locally, he also may want to find the nodes (e.g., genes/proteins) the most likely to interact with a specific node of special interest for him. In this scenario, the biologist probably wants to find the best tradeoff between the number of true interactions he will find through the experimental validation and the cost associated to this validation. The former is measured by the recall and the latter is typically proportional to the *RPP*, which suggests the use of a lift chart. In addition, if the goal is also to minimize the rate of unsuccessful validation experiments (i.e., the precision), then also looking at the PR curve might be a good idea.
- *Global analysis of the predicted network:* We may want to use the top-ranked pairs to create a new network, or to complete an already known network, for visualization or a more global analysis of its main statistics. In these cases, we need to find the best possible tradeoff between precision (not to infer wrong things) and recall (to maximize the coverage of the true network). This tradeoff can be found from a PR curve. For example, one could derive from the PR curve the lowest confidence threshold corresponding to a precision greater than 50%.

## 4. Evaluation protocols

Given a learning set *LS*_*p*_ of pairs labeled as interacting or not, the goal of the application of supervised network inference methods is to get a prediction for all pairs not present in *LS*_*p*_ (or a subset of them depending on the application). In addition, one would like to compute an estimate of the quality of these predictions as measured with any of the metrics defined in the previous section. To obtain such estimation, one could rely only on the learning set *LS*_*p*_ as nothing is known about pairs outside this set by construction.

Standard supervised classification methods are typically validated using cross-validation (CV), i.e., leaving part of the examples in the learning sample aside as a test set, training a model from the remaining examples, and testing this model on the test set (and possibly repeat this procedure several times and average). Applying CV in the context of network inference, where we have to classify pairs, needs special care (Park and Marcotte, 2011). Indeed, the predictive performance of a method for a given pair highly depends on the availability in the training data of interactions involving any of the two nodes in the tested pair. It is typically much more difficult to predict pairs with nodes for which no example of interactions are provided in the training network.

As a consequence of this, pair predictions have to be partitioned into four sets, depending on whether the nodes in the pair to predict are represented or not in the learning sample of pairs *LS*_*p*_. Denoting by *LS_c_* (resp. *LS_r_*) the nodes from *U_c_* (resp. *U_r_*) that are present in *LS*_*p*_ (i.e., which are involved in some pairs in *LS*_*p*_) and by *TS_c_* = *U_c_*\*LS_c_* (resp. *TS_r_* = *U_r_*\*LS_r_*) unseen nodes from *U_c_* (resp. *U_r_*), the pairs of nodes to predict (i.e., outside *LS*_*p*_) can be divided into the following four families:

- (*LS_r_* × *LS_c_*)\*LS*_*p*_ : predictions of (unseen) pairs between two nodes which are represented in the learning sample.
- *LS_r_* × *TS_c_* or *TS_r_* × *LS_c_*: predictions of pairs between one node represented in the learning sample and one unseen node, where the unseen node can be either from *U_c_* or from *U_r_*.
- *TS_r_* × *TS_c_*: predictions of pairs between two unseen nodes.

**Figure 4:**
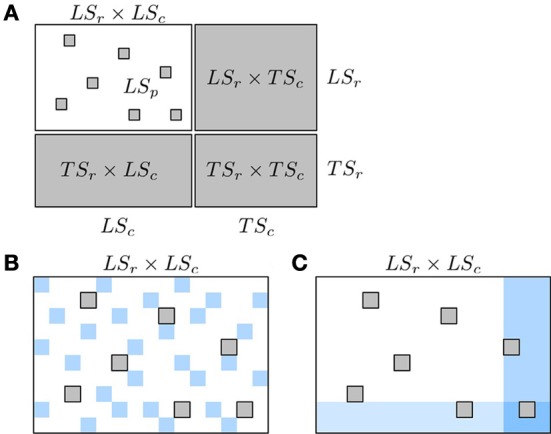
**Schematic representation of known and unknown pairs in the network adjacency matrix (A) and of the two kinds of CV, CV on pairs (B) and CV on nodes (C)**. In **(A)**: known pairs (that can be interacting or not) are in white and unknown pairs, to be predicted, are in gray. Rows and columns of the adjacency matrix have been rearranged to highlight the four families of unknown pairs described in the text: *LS_r_* × *LS_c_*, *LS_r_* × *TS_c_*, *TS_r_* × *LS_c_*, and *TS_r_* × *TS_c_*. In **(B)**,**(C)**: pairs from the learning fold are in white and pairs from the test fold are in blue. Pairs in gray represent unknown pairs that do not take part to the CV.

These pairs are represented in the adjacency matrix in Figure 4A. In this representation, the rows and columns of the adjacency matrix have been ordered, without loss of generality, in order to make nodes from *LS_r_* and *LS_c_* appear first in the ranking and as a consequence, all four groups define rectangular and contiguous subregions of the adjacency matrix. Such ordering is always possible but the respective sizes of the four groups of pairs that this ordering defines is problem dependent. Thereafter, we simplify the notations by dropping the subscript *r* and *c* and denote the prediction sets as *LS* × *LS*, *LS* × *TS*, *TS* × *LS*, and *TS* × *TS*. In the case of an homogeneous undirected graph, only three sets can be defined as the two sets *LS* × *TS* and *TS* × *LS* are confounded.

Typically, one expects different prediction performances for these different kinds of pairs and in particular, that *TS* × *TS* pairs will be the most difficult to predict since less information is available at training about the corresponding nodes. In consequence, we need ways to evaluate the quality of the predictions of these four groups separately. Below, we first present the two main CV procedures that have been proposed in the literature to evaluate supervised network inference methods and discuss which of these four kinds of predictions these procedures are evaluating (sections 4.1, 4.2). We then proceed with suggestions on how to practically assess network inference methods (section 4.3) and give an illustration on an artificial gene regulatory network (section 4.4).

### 4.1. Cross-validation on pairs

The most straightforward way to generate the learning and test sets needed for the CV, is to randomly select pairs from all the known pairs in *LS*_*p*_ (see Figure 4B). For example, in a specific step of a 10-fold CV, 90% of all the pairs from *LS*_*p*_ are chosen to be in the learning set, while the remaining 10% are then part of the test set. We call such CV *CV on pairs*. Many papers from the literature on supervised network inference only consider this sampling method (see e.g., Qi et al., 2006; Chang et al., 2010; Park and Marcotte, 2011; Yabuuchi et al., 2011).

With CV on pairs, each test set could in principle mix pairs from the four groups aforementioned. If *LS*_*p*_ is relatively dense, however, (i.e., there are only very few or no pairs in *LS_r_* × *LS_c_*\*LS*_*p*_), the chance to have a node in a test set pair not present in any learning set pair will be very low. The test set will then be largely dominated by pairs from the *LS* × *LS* group. In this case, one can thus only consider the performance evaluated by CV on pairs as representative of the performance for the *LS* × *LS* pairs. When used to assess the global performance of a method, however, CV on pairs will in general give too optimistic estimates.

To obtain an estimate of the four kinds of predictions using CV on pairs, one could partition the pairs in the test fold into the four groups and then estimate the performance for each group separately. The CV scheme proposed in the next section provides, however, a more natural way to assess the three types of predictions involving the *TS*. CV on pairs should thus be reserved for the evaluation of *LS* × *LS* pairs. For that purpose, removing pairs in the test folds that do not belong to the *LS* × *LS* group might be useful to obtain a better estimate, especially when the size of *LS*_*p*_ is small with respect to the size of *LS_c_* × *LS_r_*.

### 4.2. Cross-validation on nodes

Instead of sampling pairs, several authors have proposed to sample nodes. In the general case of a bipartite graph, the idea is to randomly split both sets *LS_c_* and *LS_r_* into two sets, respectively denoted *LS*′_*c*_ and *TS*′_*c*_ for *LS_c_* and *LS*′_*r*_ and *TS*′_*r*_ for *LS_r_*. The model is trained on the pairs in (*LS*′_*c*_ × *LS*′_*r*_) ∩ *LS*_*p*_ and then evaluated separately on three subsets (see Figure 4C):

- (*LS*′_*c*_ × *TS*′_*r*_) ∩ *LS*_*p*_ that gives an estimate of the *LS* × *TS* performance,
- (*TS*′_*c*_ × *LS*′_*r*_) ∩ *LS*_*p*_ that gives an estimate of the *TS* × *LS* performance,
- (*TS*′_*c*_ × *TS*′_*r*_) ∩ *LS*_*p*_ that gives an estimate of the *TS* × *TS* performance.

In addition, it might be interesting to evaluate the performance on the union of the three previous subsets of pairs to give an idea of the overall performance of the method. Better estimates could also be obtained by averaging results over *k* splits instead of one, where the different splits can be obtained either by repeated random resampling or by partitioning the two sets into *k*-folds and considering each fold in turn as a test set. In this latter case, partitioning *LS_c_* and *LS_r_* into *k* folds will lead to *k*^2^ candidate (*LS*′_*c*_, *LS*′_*r*_) pairs for training and (*TS*′_*c*_, *TS*′_*r*_) pairs for evaluation but one could select only *k* of them arbitrarily to reduce the computational burden. The same approach can also be applied to homogeneous graphs to obtain estimate of the *LS* × *TS* and *TS* × *TS* performances.

CV on nodes has been applied, for example, for evaluating *LS* × *TS* and *TS* × *TS* performances for the prediction of a protein–protein interaction network and an enzyme network in Kato et al. (2005), Vert and Yamanishi (2005), Geurts et al. (2007); or for evaluating *LS* × *TS*, *TS* × *LS*, and *TS* × *TS* performances for the prediction of drug-protein interactions in Yamanishi et al. (2008).

### 4.3. Discussion

CV on pairs provides a natural way to estimate *LS* × *LS* predictions, while CV on nodes provide a natural way to estimate *LS* × *TS*, *TS* × *LS*, and *TS* × *TS* predictions. A global performance assessment of a method can therefore only be obtained by combining these two protocols. This was done only by a few authors (e.g., Yip and Gerstein, 2008; Bleakley and Yamanishi, 2009; Takarabe et al., 2012). The necessity to evaluate all four groups is, however, problem dependent. Again, when designing a new supervised network inference method, it is useful to report performances for all families separately, as a method can work well for one family and less good for another. If one is interested in the completion of a particular biological network, then the need for the evaluation will depend, on the one hand, on the content of the learning sample *LS*_*p*_ and, on the other hand, on which kinds of predictions the end user is interested in. Indeed, if all nodes are covered by at least one known interaction in *LS*_*p*_, then there is no point in evaluating *LS* × *TS* or *TS* × *TS* predictions. If *LS*_*p*_ corresponds to a complete rectangular submatrix of the adjacency matrix (i.e., *LS*_*p*_ = *LS_c_* × *LS_r_*), then there is no point in evaluating *LS* × *LS* predictions. Also, for some applications, the end-user might not be interested in the extension of the network over one of the two dimensions. For example, when inferring a regulatory network, one might only be interested in the prediction of new target genes for known TFs and not in the prediction of new TF (e.g., Mordelet and Vert, 2008).

In addition to the four groups previously defined, it is also possible to evaluate independently the predictions related to each individual node (to get for example an idea of the quality of the predictions of new target genes for a given TF). This can be achieved by dividing the test folds according to one of the nodes in the pairs and then to assess performance for each partition so obtained. In practice also, the quality of a prediction depends not only on the fact that the nodes in the pair belong or not to the learning sample, but also on the number of pairs in the learning sample that concern these nodes. We can indeed expect that, for a given node, the more interactions or non-interactions are known in the learning sample for this node, the better will be the predictions for the pairs that involve this node. Assessing each node separately can thus make sense to better evaluate this effect. We will illustrate this idea in section 4.4.2.

When using *k*-fold CV to estimate ROC or PR curves, one question we have not addressed so far is how to aggregate the results over the different folds. There are several ways to do that. If one is interested only in AUROC or AUPR values, then one could simply average AUROC or AUPR values over the *k* folds. If one wants to estimate the whole ROC or PR curves, there are two ways to obtain them: first, by averaging the *k* curves to obtain a single one, second by merging pairs from the *k* test folds with their confidence score and building a curve from all these pairs. In the first case, there are several alternative ways to average ROC (and PR) curves. One of them is to sample the x-axis in each curve and then average the *k* y-axis values corresponding to these points [this is called vertical averaging in Fawcett (2006)]. Merging all predictions together is easier to implement but it assumes that the confidence scores obtained from the *k* different models are comparable, which is not trivially true for all methods. Note that our own practical experience shows that there are only very small differences between these two methods of aggregation and we usually prefer to average the individual ROC curves so that they do not have to address the question of the compatibility of the confidence scores.

Finally, we have seen in section 3.4 that PR curves depend on the ratio between positives and negatives. This dependence should be taken into account when performing CV. If CV on pairs and CV on nodes use uniform random sampling, resp. of pairs and of nodes, to define the test folds, then they implicitly assume that the ratio between positives and negatives is the same in the test fold as in the learning sample of pairs. This seems a reasonable assumption in most situations but if one expects a different ratio among the predictions, then the procedure developed in section 3.4 can be used to correct the PR curve accordingly.

### 4.4. Illustration

In this section, we will illustrate the use of CV with experiments on an artificial network. An artificial network was chosen so that it is possible to accurately estimate performance and therefore assess the different biases discussed in the paper. The chosen network is the artificial regulatory network simulated in the context of the DREAM5 network inference challenge (Marbach et al., 2012). This network is an artificial (bipartite) regulatory network, composed of 1565 genes, 178 TFs, and 4012 interactions, corresponding to 1.4% of all the pairs. The network has to be inferred from 804 artificial microarray expression values obtained in various conditions and mimicking typical real microarray compendia. To provide experiments on a homogeneous network as well, we transformed this network into a co-regulatory network composed of 1565 genes and in which there is an interaction between two genes if they are regulated by at least one common TF. The resulting network is composed of 4,191,120 interactions, corresponding to 17.1% of all pairs.

#### 4.4.1. Performance over the four families of predictions

**Figure 5:**
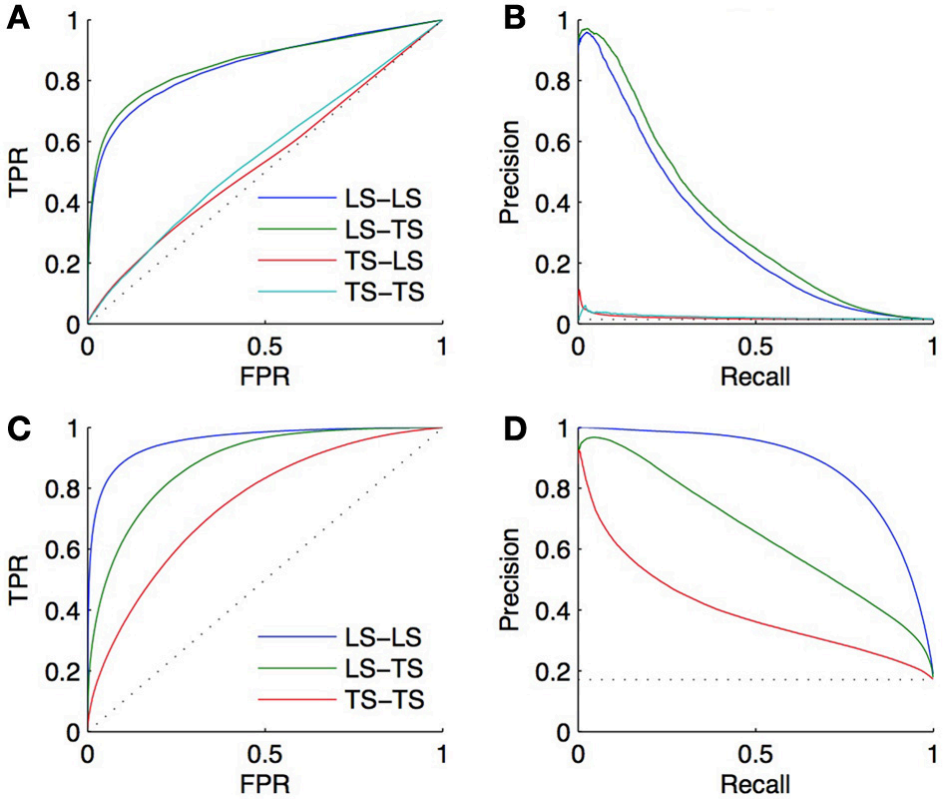
**ROC curves (A) and PR curves (B) for the four groups of predictions obtained by 10-fold CV on the DREAM5 artificial gene regulatory network**. AUROC are, respectively, equal to 0.85, 0.86, 0.53, and 0.55 and AUPR are equal to 0.31, 0.34, 0.02, and 0.02. The performance of prediction of a pair involving a gene and a TF present in the learning set (*LS* × *LS*) is as good as the performance of prediction of a pair involving a gene absent and a TF present in the learning set (*LS* × *TS*). On the contrary, predicting an interaction involving a new TF is much more difficult (*TS* × *LS* and *TS* × *TS*). Bottom: ROC curves **(C)** and PR curves **(D)** obtained by 10-fold CV on the corresponding DREAM5 co-regulatory network. AUROC are, respectively, equal to 0.96, 0.88, and 0.75 and AUPR are equal to 0.88, 0.65, and 0.40. Predictions on pairs involving two genes from the learning set are the best, while predictions on pairs involving two genes from the test set are the worst.

We performed a 10-fold CV on both the bipartite and homogeneous networks, with a local approach using Random Forests (Breiman, 2001). For the bipartite network, we sample first on pairs, and second on genes and on TFs. The resulting curves and areas under the curves are given in Figures 5A,B. Surprisingly, the prediction of interactions involving a TF present in the learning set, and a new gene (*LS* × *TS*) gives slightly better scores than the prediction of interactions involving a gene and a TF both present in the learning set (*LS* × *LS*). On the other hand, the prediction of pairs involving a new gene and a TF present in the learning set (*LS* × *TS*) or not (*TS* × *TS*) gives performances barely better than random. Finding new interactions for a known TF is thus much easier than finding interactions for a known gene.

For the homogeneous network, we sample first on the pairs and second on the genes. The resulting curves are shown in Figures 5C,D. Prediction of coregulation between two genes belonging to the learning set gives the best AUROC and AUPR. As expected prediction of coregulation between one known gene and one new gene gives less good performance, followed by prediction of coregulation between two new genes.

These two examples clearly highlight the fact that all pairs are not as easy to discover as the others, and that it is thus important to distinguish them during the validation.

#### 4.4.2. Per-node evaluation

**Figure 6:**
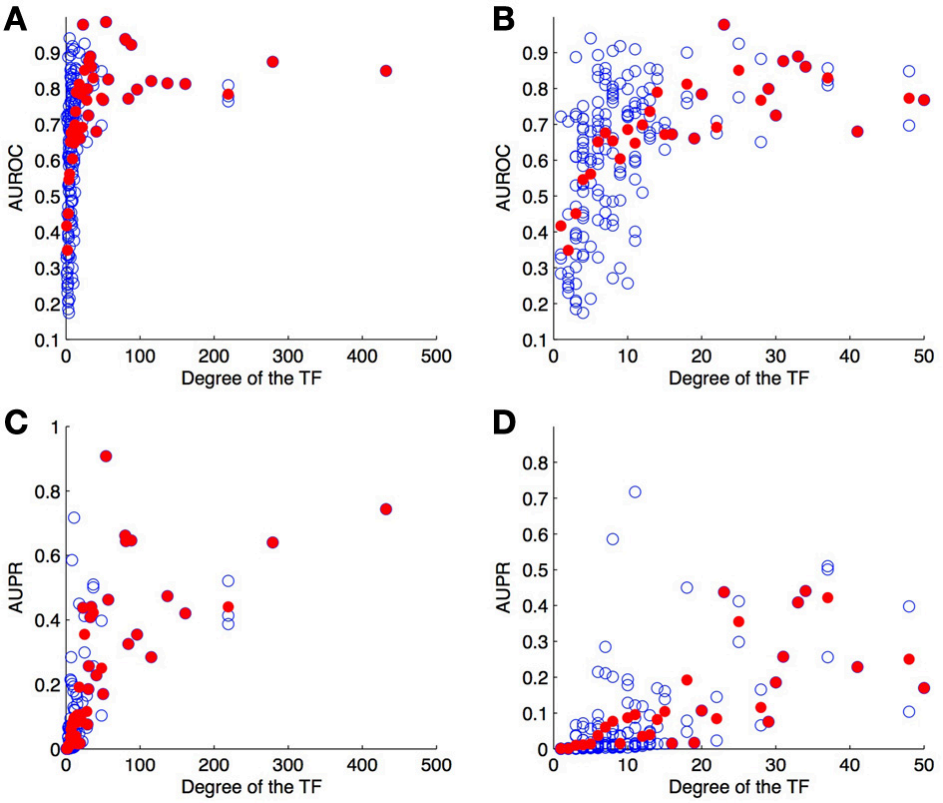
**AUROC (A,B) and AUPR (C,D) for each TF as a function of its degree (number of targets) on the DREAM5 network**. Each value was obtained by 10-fold CV on genes. Each blue point corresponds to a particular TF and plots its average AUROC or AUPR value over the 10-folds. Each red point correspond to the average AUROC or AUPR values over all TFs of the corresponding degree. Globally, the higher the degree, the higher are the areas under the curve and so the better are the predictions.

As a second experiment, we computed the ROC and PR curves for each of the 178 TFs separately, from the result of the 10-fold CV on genes (bipartite graph). Figure 6 shows the (average) AUROC and AUPR values for all TFs according to their degree. This plot shows that the quality of the predictions differs greatly from one TF to another and that the number of known pairs seems to affect this quality. For low values of degree (lower than about 20), the AUROC globally increases when the degree increases, but for higher values the AUROC does not seem to depend on it. On the other hand, AUPR values globally increase when the degree increases, for all values of TF.

#### 4.4.3. A more realistic setting

The goal of CV is to estimate, from the training subnetwork, the performance one expects on the prediction of new interactions. We carried out a last experiment to evaluate the quality of the estimation obtained by CV in a realistic setting. In this setting, we assume that the known pairs are obtained by first randomly drawing 2/3 of the genes and 2/3 of the TFs and then randomly drawing 2/3 of all interacting and non-interacting pairs between these genes and TFs. The resulting training set thus contains about 30% of all possible pairs and the goal is to predict the remaining 70% pairs, which are divided into, respectively 15%, 22%, 22%, and 11% of *LS* × *LS*, *LS* × *TS*, *TS* × *LS*, and *TS* × *TS* pairs.

**Figure 7:**
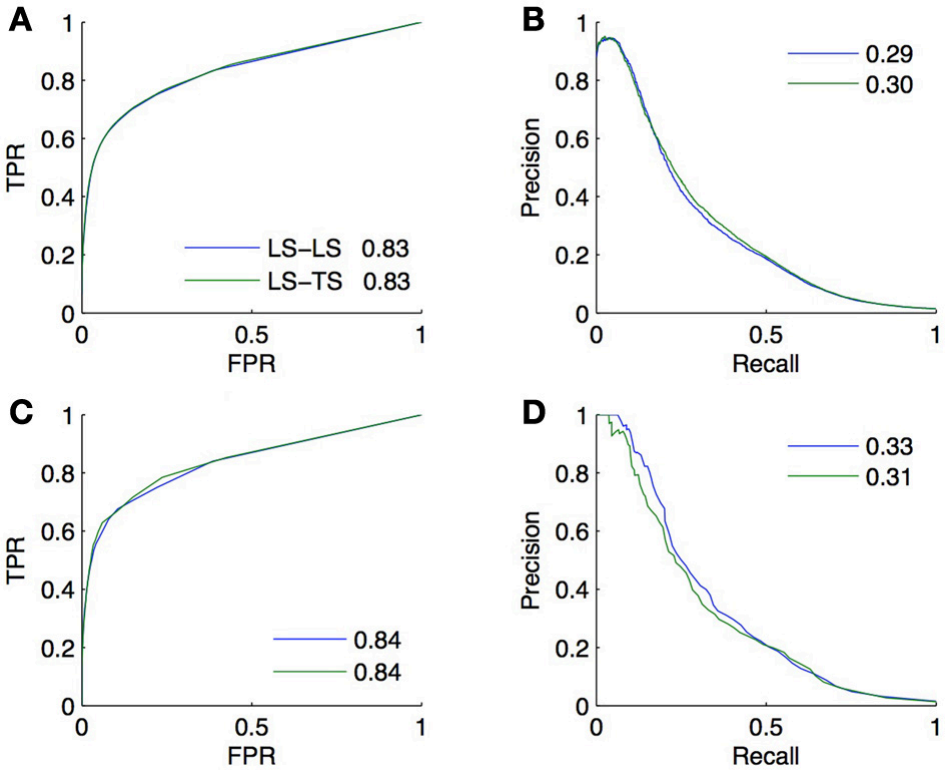
**Comparison of the CV estimates of the *LS* × *LS* and *LS* × *TS* scores, ROC curve in (A) and PR curve in (B), with true score values for the same two families of predictions, ROC curve in (C) and PR curve in (D)**. AUROC and AUPR values are found in the legends.

Two validation experiments were performed. First, we evaluated the performance of the (global) Random Forests method by CV across pairs and across nodes on the 30% of known pairs (experiment A). Second, we trained local models based on Random Forests on the known pairs and we evaluated them on the 70% of pairs not used during training. Experiment A is therefore supposed to provide a CV estimate of the true performance as estimated by experiment B. The resulting ROC and PR curves obtained from these two experiments for the *LS* × *LS* and *LS* × *TS* families are shown in Figure 7. As expected, for both kinds of predictions, the curves obtained by the two experiments are very similar, with a very slight advantage to experiment B. This small difference comes from the fact that the number of pairs in the learning set of experiment B is 10% greater than the number of pairs in the learning sets of experiment A (because of 10-fold CV).

## 5. Lack of negative examples

In biological networks, often truly non-interacting pairs are not available. Indeed it is often impossible for biologists to experimentally support the lack of an interaction between two nodes. For example you can prove that a specific drug acts on a set of proteins, and you may want to find other proteins being affected by this drug by using machine learning techniques, but you cannot prove that a particular set of proteins is not affected by the drug. This lack of negative examples leads to problems both when training and when evaluating a model. We discuss these two steps separately below and conclude with an illustration.

### 5.1. Training a model

Standard supervised machine learning methods require both positive and negative examples for training. The most common way to get around this limitation in the presence of only positive examples is to take as negative examples all, or a subset of, the unlabeled examples, i.e., in our context, considering all or some pairs that have not been measured as interacting as actually non-interacting. This approach has been adopted by most authors in the literature, e.g., in Geurts et al. (2007), Mordelet and Vert (2008), Yamanishi et al. (2008), Yip and Gerstein (2008), Bauer et al. (2011), van Laarhoven et al. (2011), and Takarabe et al. (2012), the authors use all unlabeled pairs as negatives and in Yip and Gerstein (2008), Chang et al. (2010), Hue et al. (2010), Yabuuchi et al. (2011), and Yu et al. (2012) they use only a subset of them. Although there is a risk that the presence of false negatives in the learning sample will affect the performance of the machine learning method, using only a subset of the unlabeled pairs as negative examples will, however, substantially reduce this risk in the context of biological networks. Indeed, the fraction of positive interactions is expected to be very small in common biological networks, which will lead to only a very small number of false negatives in the learning sample as soon as the size of the negative set is not too large with respect to the size of the positive set. For example, for the protein–protein interaction network of the yeast, it is estimated that 1 pair over 600 is actually interacting (Qi et al., 2006), which corresponds to ~0.2% of all the possible pairs. A learning sample composed of 1000 positive and 1000 unlabeled pairs is therefore expected to contain in average only about two or three false negatives. In addition to the reduction of the number of false negatives, sampling the unlabeled pairs has also the advantage of decreasing the computational cost at the training stage and of improving the class imbalance in the training sample, which might affect the performance of classification methods (Pandey et al., 2010; Park and Marcotte, 2011).

To even further reduce the risk of incorporating false negatives in the training data, one could also replace random sampling from the unlabeled pairs by a selection of a subset of more reliable negative examples using prior knowledge about the biological interactions of interest. This approach was considered for example in Ben-Hur and Noble (2006) for protein–protein interactions, in Ceccarelli and Cerulo (2009) for gene-TF interactions, and in Yousef et al. (2008) for microRNA-gene interactions.

Note that the presence of false negatives is not necessarily detrimental. Elkan and Noto (2008) showed that, under the assumption that the interactions in the learning sample are selected uniformly at random among all interactions, the presence of false negatives in the learning sample will only affect the confidence scores by a constant factor, which will thus leave ROC and PR curves for example unaffected. Although their assumption is quite strong, this nevertheless suggests that the presence of false negatives might have just a marginal effect on performance. As an illustration, we run the same experiment as in section 4.4 on the DREAM5 regulatory network only turning 10% of positives into negatives when training the model. The AUPR reduces from 0.31 to 0.29 and the AUROC from 0.85 to 0.84, showing that the presence of false negatives only very slightly affects the performance of Random Forests.

One drawback of considering unlabeled pairs as negative pairs for training the model is that the predictions provided by the model for these pairs will be biased toward low confidence scores. One way to obtain unbiased predictions for all unlabeled pairs is to use CV: construct a model using all known positive pairs and a random subset of the unlabeled pairs as negatives, use this model to obtain a prediction for all unlabeled pairs not used during the training stage, and repeat the procedure several times using different subsets of unlabeled pairs until all unlabeled pairs have obtained at least one prediction. Based on this general scheme, Mordelet and Vert (2013) proposed to train several models using small random subsamples of unlabeled pairs, leading to several predictions for each unlabeled pairs that are then aggregated.

Another approach to deal with the lack of negative examples is to forget about unlabeled examples and exploit machine learning methods, such as one-class support vector machines (Schölkopf et al., 2001), that can learn a model only from the positive examples. This approach was for example adopted in Yousef et al. (2008) to predict miRNA-gene interactions. Machine learning literature also provides several specific algorithms for dealing with positive and unlabeled examples, among which for example (Lee and Liu, 2003; Denis et al., 2005; Geurts, 2011), that could also be used in the context of supervised network inference. Geurts (2011) validated his method for the inference of regulatory networks, which showed improvement over standard two-classes methods.

### 5.2. Evaluating a model

The absence of true non-interacting pairs in the training data has also an impact on the validation of the model, as the different evaluation measures described in section 3 all rely on the availability of a set of known interacting and non-interacting pairs on which to perform the CV.

Like for training, the simplest way to deal with the lack of negatives for validating the model is to consider all unlabeled pairs within the test folds (generated in the context of CV on pairs or CV on nodes) as non-interacting pairs and then estimate ROC or PR curves under this assumption. The presence of false negatives in the gold standard will obviously affect the estimation of the performance. Let us assume that the ranking of the examples in a test fold is fixed and that a proportion *x* of positives are turned into negatives. Under this assumption, it can be shown that the *TPR* remains unchanged while *FPR* and *Prec* are modified as follows:

(1) $${\text{FPR}}_{\text{new}}=\frac{FP+TP\cdot x}{N+P\cdot x}>\text{FPR}$$

(2) $${\text{Prec}}_{\text{new}}=(1-x)\text{Prec}<\text{Prec},$$

where the first inequality holds as soon as the ranking is better than random (see the supplementary information for the details). One can thus expect that the introduction of false negatives will systematically degrade both the ROC and the PR curves.

**Figure 8:**
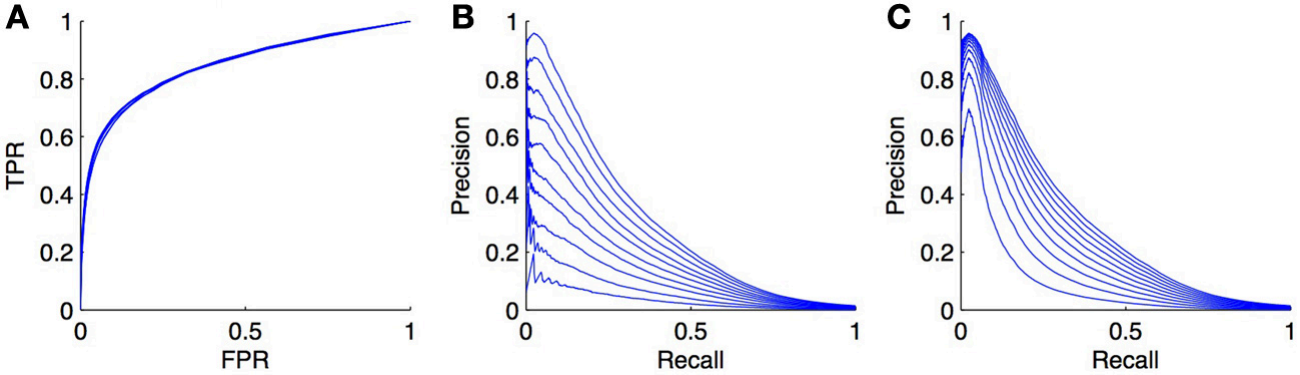
**Effect of false negatives on ROC and PR curves**. We simulated false negatives in the DREAM5 regulatory network, during the testing stage. The ratio of false negatives does not influence the ROC curve **(A)**, but the PR curve **(B)** decreases while the ratio of positives turned into negatives increases. The ratio varies from 0 to 0.9. Curves **(C)** show the evolution of the PR curve when the ratio *P*/*N* is set similarly as in **(B)**. Although the PR curve degrades also in this case, the degradation is not as important as when false negatives are introduced.

As an illustration, we carried out simulations on the DREAM5 regulatory network (see section 4.4). The model was trained with Random Forests with the local approach and we focus our experiment on the *LS* × *LS* pairs. The learning sample was kept unchanged but in each of the 10 CV folds (CV on pairs), we randomly turned a fraction *x* of positives into negatives, in order to simulate the introduction of false negatives. We tried several proportions *x* ∈ {0, 0.1, 0.2, …, 0.9} and got the curves shown in Figures 8A,B. As expected, the PR curves degrade when the ratio increases. More surprisingly, the ROC curves do not seem to be influenced by the ratio of false negatives. This can be explained by the fact that in Equation (1), *TP* · *x* becomes negligible compared to *FP* and *P* · *x* is negligible compared to *N*, even for small *FPR* values as soon as *N* is large with respect to *P*.

Actually, there are potentially two effects that play a role in the degradation of the PR curve in Figure 8B: the introduction of false negatives but also the alteration of class imbalance. Indeed, we have seen in section 3.4 that the PR curve was affected by this ratio. To try to assess both effects separately, we also generated the PR curves obtained from the initial curve by increasing the number of negatives in such a way that the ratio of *P*/*N* matches the ratio of *P*/*N* in the previous experiment for *x* ranging from 0 to 0.9. These curves are plotted in Figure 8C. They are also systematically degraded by the introduction of more negatives but the degradation is not as high as the degradation obtained by the addition of false negatives.

We can conclude from these experiments that PR curves are much more sensitive than ROC curves to false negatives in the true dataset. Interestingly, given Equation (2), if we can estimate the ratio *x* of false negatives, we can modify the PR curve simply by dividing the precision by 1 − *x*, to obtain a more realistic PR curve. Note, however, that the correction in Equation (2) only applies under the assumption that false negatives will get scores distributed similarly as positives. This assumption is not unrealistic in practice as we indeed expect that false negatives will be predicted most often as positives (since they are in fact positives). However, it is also possible that for a given biological network, known interactions are the strongest ones (i.e., those with the strongest experimental support) and therefore false negatives will typically correspond to weaker interactions. Their scores, as predicted by network inference methods, can then be smaller than those of known positives. In this case, the degradation of the PR curve will most probably be somewhere in between curves in Figures 8B,C. Note that even though PR curves are affected by the introduction of false negatives, this is not really problematic when it comes to compare different inference methods on the same networks, as all methods will be affected in the same way by these false negatives. In this case, correcting the PR curve is not necessary.

Finally, we would like to note that the ratio between positives and negatives used to evaluate PR curves should be as close as possible to the expected ratio in the pairs to predict. Indeed, one could be tempted to estimate performance by CV on pairs on the positives and the selected negatives (randomly or from prior knowledge). The resulting PR curves will be, however, representative only for the given observed ratio between positives and negatives. If this ratio is different from the expected one, then one should apply the PR curve correction presented in section 3.4.

### 5.3. Illustration

To illustrate the practical impact of the absence of negatives on validation, we reproduced the experiment of section 4.4.3 on the DREAM5 network, this time assuming that only positive (and unlabeled) pairs are available in the training data. More concretely, we again first randomly drew 2/3 of the genes and 2/3 of the TFs and then randomly drew 2/3 of the positive pairs existing among these genes and TFs. This set of positive pairs then defines our training network and the goal is to find new positive pairs among all the other ones (that are then considered as unlabeled). The positive pairs in the training set were chosen so that they match the positive pairs in the training set in the experiment of section 4.4.3.

**Figure 9:**
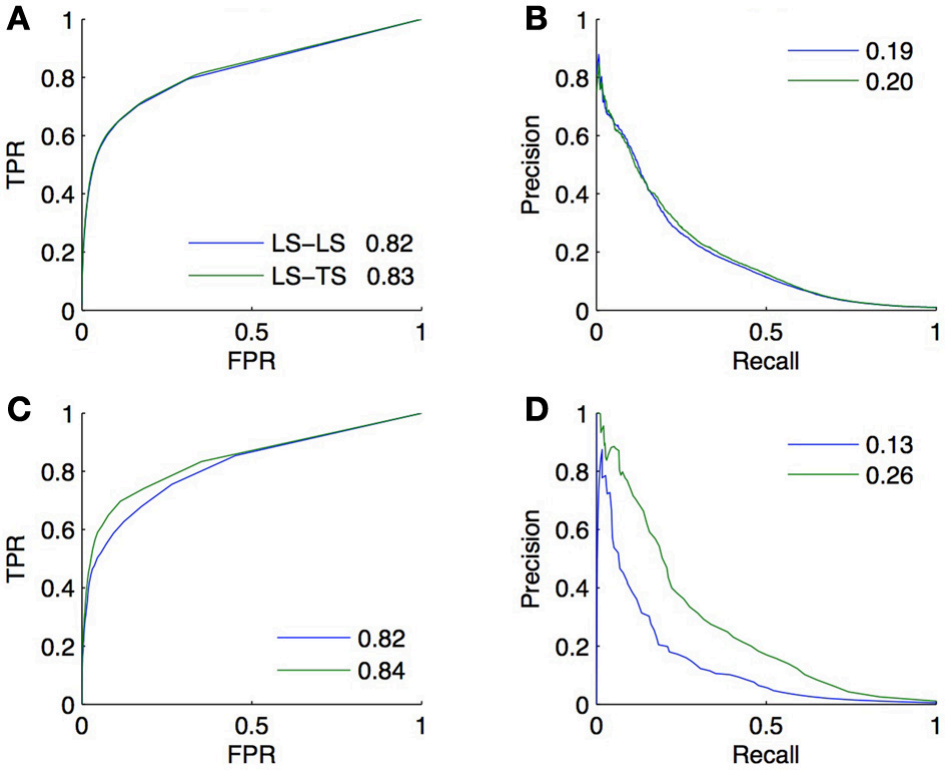
**Comparison of the CV estimates of the *LS* × *LS* and *LS* × *TS* scores, ROC curve in (A) and PR curve in (B), with true score values for the same two families of predictions, ROC curve in (C) and PR curve in (D), when only positive and unlabeled pairs are available**. AUROC and AUPR values are found in the legends.

Two validation experiments were performed. First, CV across pairs and nodes was carried out on all pairs between the selected genes (2/3) and TFs (2/3), considering all unlabeled pairs as negative (experiment A). Second, we randomly split the whole set of unlabeled pairs into two subsets. We trained a model on the positive pairs and each of these subsets taken in turn as the set of negative pairs and then used this model to obtain a prediction for the unlabeled pairs in the other subset. The resulting predictions were then evaluated against the true network (experiment B). Experiment A is thus supposed to provide a CV estimate of the true performance as computed by experiment B. The resulting ROC and PR curves obtained from these two experiments are shown in Figure 9 for the *LS* × *LS* and *LS* × *TS* families.

ROC curves and AUROC scores obtained from experiments A and B are very close but noticeable differences appear in PR curves and AUPR scores. Indeed, experiment A gives higher AUPR than experiment B for *LS* × *LS* pairs, but gives lower AUPR for *LS* × *TS* pairs. In other words, CV overestimates the AUPR for *LS* × *LS* pairs and underestimates it for *LS* × *TS* pairs. As discussed above, these differences can be explained, on the one hand, by the presence of false negatives in the test data generated by the CV and, on the other hand, by the differences in the ratio between positives and negatives that exist in the two families of pairs between experiments A and B.

Assuming that both the ratio of false negatives in the training pairs and the ratio of positives and negatives among the unlabeled pairs are known or can be estimated, PR curves and AUPR scores obtained from experiment A can be corrected using results in sections 3.4, 5.2, so that they match the conditions of the application of the model in experiment B. Since these quantities are known for our artificial network, we performed these corrections, first adjusting the precision to account for the false negatives and then correcting the curve to account for the different ratio of positives versus negatives. The corrected AUPR are respectively 0.16 and 0.26 for *LS* × *LS* and *LS* × *TS*, which are now closer to the value obtained from experiment B.

Note that another factor that could introduce a difference between CV scores and real scores is the composition of the training data in terms of positives and negatives, which might affect learning algorithms. In our experiment, however, the ratios of positives versus negatives in the training data are very close (~ 0.9% for experiment A and ~ 1.0% for experiment B).

## 6. Impact of heavy-tailed node degree distribution

Biological networks are typically non-random. In particular, many of them have a heavy-tailed distribution of node degrees: several nodes, called hubs, have degrees greatly higher than the average (Stumpf and Porter, 2012). In such networks, a new node, without consideration of its features, is more likely to interact with a hub than with a less connected node. As a consequence, it is possible in such network to obtain better than random interaction predictions without exploiting the node features, by simply connecting any new node with the more connected nodes in the training network.

**Figure 10:**
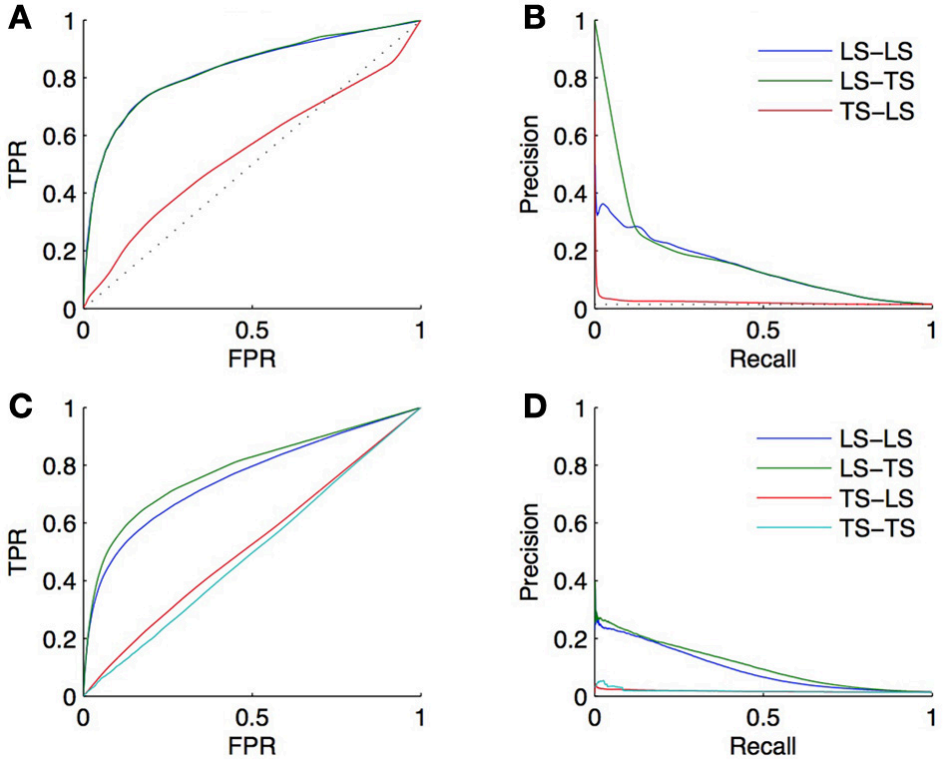
**The heavy-tailed degree distribution of many biological networks can lead to better than random predictions, only by exploiting the network topology and ignoring node or pair features**. First row: ROC curves **(A)** and PR curves **(B)** obtained from predictions made on the DREAM5 dataset using the degree of the nodes in the learning set. Second row: ROC curves **(C)** and PR curves **(D)** obtained from predictions made on the DREAM5 dataset when randomly permuting the feature vectors relative to different nodes.

Let us illustrate this on the DREAM5 *in silico* network. The topology of this network is based on known transcriptional regulatory networks of model organisms such as *S. cerevisiae* and *E. coli*. It clearly has a heavy-tailed node degree distribution (5% of the TFs collect about 50% of all interactions). Figures 10A,B shows the ROC and PR curves obtained using the same 10-CV folds as in section 4.4.1. The *LS* × *LS* pairs are now ranked according to the sum of the degrees of the nodes, computed in the training network, and the *LS* × *TS* and *TS* × *LS* pairs are now ranked according to the degree of the TF and of the gene, respectively. The AUROC and AUPR are, respectively, equal to 0.83 and 0.14 for *LS* × *LS*, 0.83 and 0.17 for *LS* × *TS*, and 0.54 and 0.02 for *TS* × *LS*. We can conclude from these results that the degree of a TF is indeed greatly linked with the probability for it to interact with a known or a new gene. On the contrary, the degree of a gene does not influence its chance to interact with a new TF. Although better than random, it is important to note, however, that the degree-based ranking of *LS* × *TS* pairs does not allow to distinguish potential targets of a given TF since they all inherits the degree of the TF.

That it appears possible to complete a network based only on the degree of *LS* nodes shows that using a random classifier as a baseline for assessing the performance of supervised network inference methods is inappropriate. A network inference method that does not perform better than the simple degree-based ranking of the interactions is potentially unable to effectively extract useful information from the features. As a consequence, we believe that one should always report the performance of the degree-based ranking as a baseline for assessing the performance of a supervised network inference method. As an illustration, on the DREAM5 network, we obtained with the Random Forests method AUROC values of 0.85 and 0.86 and AUPR values of 0.31 and 0.34, respectively for *LS* × *LS* and *LS* × *TS* pairs (see section 4.4.1). The AUROC values of 0.85 and 0.86, although very good in absolute values, should be treated cautiously; they are indeed only slightly greater than the 0.83 AUROC of the degree-based ranking. In contrast, the doubling of the more robust AUPR value (from 0.14 and 0.17 for the degree-based random predictor to 0.31 and 0.34 for the trained model) indicates that the Random Forests are able to capture information from the feature vectors and indeed enable reliable predictions.

Even when the features are uninformative, supervised inference methods should be in principle able to “learn” and exploit this positive bias for interactions with nodes of high degree within the training data. Indeed, this is in this case the only way to get non-random predictions. To illustrate this assumption, we carried out an experiment on the DREAM5 network with the same protocol as in section 4.4.1 but making the features uniformative. To decorrelate the features from the network, the model is trained and tested by 10-fold CV on new data obtained by keeping the labels of the pairs unchanged but randomly permuting the feature vectors of the nodes. Resulting ROC and PR curves for *LS* × *LS* and *LS* × *TS* pairs are shown in Figures 10C,D. The AUROC and AUPR are, respectively, equal to 0.76 and 0.09 for *LS* × *LS* and 0.78 and 0.11 for *LS* × *TS*. These results are slightly worse than the results obtained by the degree-based ranking but they are much better than random, although the features do not convey any information about the network by construction. Note that the AUROC and AUPR values averaged over each TF (as done in section 4.4.2) are, respectively, equal to 0.48 and 0.02 for *LS* × *TS* pairs. Like the degree-based ranking, the model trained on permuted features is unable to distinguish between possible targets of a given TF. This latter experiment further confirms that the degree-based ranking should be preferred to a random ranking as a baseline to assess the performance of supervised network inference methods.

## 7. Discussion

In this paper, we discussed measures and protocols for the validation *in silico* of supervised methods for the inference of biological networks, i.e., methods that infer a biological network from a training sample of known interacting and non-interacting pairs and a set of features defined on the network nodes (or directly on pairs of nodes). Although this problem is very close to a standard supervised classification problem, it requires to address several important issues related to the need to classify pairs of entities in a candidate interaction and to the nature of biological networks. We carried out a rigorous examination of these issues that we supported by experiments on an artificial gene regulatory network. The main guidelines that can be drawn from this examination are as follows:

- Network inference methods have been assessed mainly using PR curves and ROC curves. The choice of an appropriate metric should be dictated mainly by the application but generally PR curves are more appropriate than ROC curves given the highly imbalanced nature of the underlying classification problem, related to the very sparse nature of most biological networks. While PR curves are sensitive to the ratio of positives versus negatives in the test data, we show that it is straightforward to adapt them to a new ratio. A further important characteristic of biological networks that should influence the choice of a performance metric is the heavy-tailed degree distribution. We show that this degree distribution severely affects the ROC curves, making it difficult to estimate the performance of inference methods by the AUROC, while PR curves are much less affected.
- When validating a model, it is necessary to divide the predictions into four groups, given that the two nodes might either be present or absent in the learning sample of interactions. Indeed, performance is typically very different from one group to another and improves when the number of training interactions involving the nodes in the pairs to be predicted increases. The quality of the predictions for pairs where both nodes have interactions in the training network can be assessed using CV over pairs in the training data. The quality of the predictions for the three other groups of pairs, where at least one node is not represented in the training data, is best assessed by using CV over nodes. Unless the inference problem at hand makes some subgroups of predictions irrelevant, we advocate the joint use of both kinds of CV to get a more detailed assessment of the performance of an inference method.
- We discussed the lack of experimental support for non-interacting pairs in most biological networks. We reviewed several ways to address this problem at training time and showed that the presence of false negatives does not really affect ROC curves but can result in an underestimation of the PR curve. Assuming that the proportion of false negatives in the test data is known and that false negatives are selected randomly among positives, we show that it is possible to correct the PR curve so that it better reflect true performances. The correction is, however, not necessary when one only wants to compare different methods.
- We showed empirically that a heavy-tailed node degree distribution seemingly enables a better than random inference only by exploiting the topology of the training network. As a consequence, random guesses should not be taken as valid baselines for supervised network inference methods, in order not to overestimate the performance. Every validation of a supervised inference method should always be supplemented by a reporting of the performance of the simple degree-based score (or a classifier grown from randomly permuted feature vectors).

Thereby, we provided the most comprehensive examination and discussion of issues in the evaluation of supervised inference techniques so far. Given that the examined supervised techniques exploiting prior information on the network are typically superior in performance to unsupervised approaches, a reliable assessment is particularly desirable. Following the guidelines we derived will enable a more rigorous assessment of supervised inference methods, will contribute to an improved comparability of the different approaches in this field and will thus furthermore aid researchers in improving the state of the art methods.

Still, there remain several open questions about supervised network inference methods and their validation. First, with a few exceptions, most papers in the domain focus on a given type of biological network. Yet, unlike unsupervised methods that need some prior knowledge to derive their confidence scores, supervised methods are most of the time generic in that they could be applied to any network without much adaptation. A thorough empirical comparison of these methods on several networks with different characteristics is missing to really understand the advantages and limitations of all these methods. While we argue, as others, that predictions within the different pair subgroups should be assessed separately, we have not discussed ways to take into account the resulting information to obtain better global network predictions. Indeed, most methods eventually provide a single ranking of all pairs to be predicted. How to take into account the performance differences between the different groups of pairs to reorganize this ranking into a better one, and whether this is actually possible, remains an interesting open question for future research. In this review, we focus on the statistical and *in silico* validation of network inference methods using CV techniques. Such validation helps assess the quality of the predictions and therefore decide on a confidence threshold that best suits application needs. However, even more important is the experimental validation of the predictions provided by network inference techniques. Experimental validation depends on the nature of the biological network at hand and therefore a discussion of these techniques is out of the scope of this review. Note nevertheless that experimental validation will be influenced also by the lack of experimental support for non-interacting pairs and that for some (more abstract) networks, experimental validation might be very difficult (e.g., disease-gene networks).

### Conflict of interest statement

The authors declare that the research was conducted in the absence of any commercial or financial relationships that could be construed as a potential conflict of interest.
