# Supplementary material for: On protocols and measures for the validation of supervised methods for the inference of biological networks
Source: Front Genet. 2013 Dec 3;4:262. doi: 10.3389/fgene.2013.00262 (PMC3848415; doi:10.3389/fgene.2013.00262)
Supplement: Supplementary file 1 [file DataSheet1.PDF]

On protocols and measures for the validation
of supervised methods for the inference of biological networks

SUPPLEMENTARY INFORMATION

Section 3.4

Adapting PR curve to another ratio P/N

It is possible to adapt a given PR curve to another ratio between positives and negatives than the one adopted to generate it (Hue *et al.*, 2010). Let us assume a PR curve estimated from P_1 positives and N_1 positives and let us estimate from this curve a new curve corresponding to $P_2 = P_1$ positives and $N_2 \neq N_1$ negatives respectively drawn from the same distribution as the original P_1 positives and N_1 negatives. For a given confidence threshold, the recall (i.e., the proportion of positives greater than the threshold) is unchanged since $P_1 = P_2$. Denoting by TP_1 and TP_2 (resp. FP_1 and FP_2) the number of positives (resp. negatives) with a score greater than the threshold in both cases, we have $precision_1 = \frac{TP_1}{TP_1+FP_1}$ and $precision_2 = \frac{TP_2}{TP_2+FP_2}$. Given that $TP_1 = TP_2$ and $FP_2 = \frac{N_2}{N_1} FP_1$ in average, it is easy to show that $precision_1$ and $precision_2$ are related as follows:

$$precision_2 = \frac{precision_1}{precision_1 + \frac{N_2}{N_1}(1 - precision_1)}. \quad (1)$$

Using this formula, one can thus approximate the PR curve for an arbitrary ratio between positives and negatives from the knowledge of at least one PR curve (corresponding to an arbitrary ratio). Note that this PR curve can also be derived from a ROC curve and the knowledge of the actual ratio $\frac{P}{N}$ using the fact that, for any confidence threshold, we have:

$$precision = \frac{TPR}{TPR + FPR\frac{N}{P}}, \quad (2)$$

and that both TPR and FPR are known from the ROC curve.

Section 5.2

Effect of false negatives on evaluation

To estimate the effect of false negatives on the PR and ROC curves, we suppose that the ranking of the examples in a test fold is fixed and then compute the change in PR and ROC curve when a

proportion x of positives are turned into negatives. The assumption under this model is that false negative examples will get confidence scores distributed similarly as scores of positive examples. We will discuss the relevance of this assumption below.

Given this modification, the error counts in the confusion matrix are modified as follows, in average and for a given confidence thresholds (using the notations of Section 3):

$$
\begin{aligned}
P_{new} &= P - P \cdot x = (1-x)P & N_{new} &= N + P \cdot x \\
TP_{new} &= TP - TP \cdot x = (1-x)TP & FP_{new} &= FP + TP \cdot x \\
FN_{new} &= FN - FN \cdot x = (1-x)FN & TN_{new} &= TN + FN \cdot x
\end{aligned}
$$

From these changes, we can compute the resulting variations in TPR, FPR, and precision that define ROC and PR curves:

$$TPR_{new} = \frac{TP_{new}}{P_{new}} = \frac{(1-x)TP}{(1-x)P} = TPR \tag{3}$$

$$FPR_{new} = \frac{FP_{new}}{N_{new}} = \frac{FP + TP \cdot x}{N + P \cdot x} > FPR \tag{4}$$

$$Prec_{new} = \frac{TP_{new}}{TP_{new} + FP_{new}} = \frac{(1-x)TP}{(1-x)TP + FP + TP \cdot x} = (1-x)Prec < Prec \tag{5}$$

Inequality (4) is valid as soon as the ranking is better than random. Indeed, it can be shown by some straightforward manipulations that inequality (4) is equivalent to the following inequality:

$$\frac{TP}{FP} > \frac{P}{N},$$

which is verified for any classifier that is better than random (i.e., a classifier that puts more positives above the confidence threshold than expected at random).
